# An investigation into academic burnout, enjoyment and engagement in EFL learning among Chinese junior high school students

**DOI:** 10.3389/fpsyg.2023.1292772

**Published:** 2024-06-21

**Authors:** Xiaohong Zhang, Jian Wang, Xinli Ke

**Affiliations:** ^1^School of Foreign Languages and Cultures, Panzhihua University, Panzhihua, China; ^2^School of Foreign Languages and Cultures, Geely University of China, Chengdu, China; ^3^Department of Foreign Languages, Southwest Jiaotong University Hope College, Chengdu, China

**Keywords:** learning engagement, burnout, foreign language enjoyment, junior high school students, EFL learning

## Abstract

With the booming of Positive Psychology, a growing scholarly interest has emerged in language learners’ psychological well-being. However, limited research has been conducted on the emotions of Chinese EFL learners. Therefore, this study aimed to quantitatively examine the burnout, enjoyment, and engagement levels among 387 junior high school EFL learners. The findings revealed that the students experienced low levels of burnout but high levels of enjoyment and engagement in learning. Furthermore, significant gender and grade differences were observed in these variables. The study also found strong negative associations between burnout and both enjoyment and engagement, along with significant positive correlations between enjoyment and engagement. Additionally, it was discovered that enjoyment, rather than burnout, significantly predicted English learning engagement. Interestingly, the study also revealed that enjoyment fully mediated the relationship between engagement and burnout. These findings highlight the importance of promoting enjoyment in order to reduce burnout and foster increased engagement among language learners. This article concludes with the theoretical and pedagogical implications for EFL instruction.

## Introduction

Second language acquisition (SLA) research has primarily revolved around learners’ cognitive processes in language learning (Graesser et al., 2014). Various aspects of language learners’ cognitive processes have been examined, including how they attend to, process, comprehend, retain, and retrieve language knowledge (McLaughlin et al., 1983; VanPatten, 1990; Goh, 2000; Barcroft, 2007; Rott, 2007; Durrant and Schmitt, 2010). However, cognitive perspectives fall short in fully explaining language learning processes, particularly in instances where learners struggle to apply their language knowledge despite possessing the required skills (Bereiter and Scardamalia, 1985). In this regard, an integrative framework is needed that incorporates both cognitive and non-cognitive factors, such as learners’ emotional investment, to explicate the learning process (Artino et al., 2012; Boughoulid, 2023).

In previous decades of applied linguistic research, learner emotions were considered as “irrational factors,” overshadowed by cognition (Dewaele et al., 2019, p. 1). The Affective Filter Hypothesis (Krashen, 1985) gradually brought emotions in language learning to the fore (Dewaele, 2005). Nonetheless, empirical studies have predominantly focused on L2 anxiety (Dewaele and Li, 2020). Recently, SLA research has experienced an “affective turn” due to the flourishing development of Positive Psychology (PP), expanding the scope of research on learner emotions (Pavlenko, 2013). This reorientation has led to an increased scholarly interest in affective factors related to language learning (Li, 2021; Xia and Chen, 2022). L2 researchers have begun to acknowledge the diversity and significance of emotional experiences among language learners (Dewaele and Li, 2020). Instead of solely concentrating on negative emotions like foreign language (FL) anxiety, the academic community has started exploring multifarious emotional experiences in FL learning, including foreign language enjoyment (FLE), boredom, grit, burnout, guilt, shame, and more (Dewaele and MacIntyre, 2014; Teimouri, 2018; Li, 2020a). Research indicates that positive emotions enhance language learners’ performance and engagement, while negative emotions have the opposite effect (Bakır-Yalçın and Usluel, 2023; Wang et al., 2023).

We investigated the achievement emotions (i.e., burnout and enjoyment) and academic engagement of junior high school students learning English in Chinese mainland. China is home to an extensive English learning community and English learners have to sit for two significant English exams in their academic journey. In the 9th grade, they take the *Zhongkao*, a province-level senior high school entrance examination. This exam is highly competitive and determines whether students can access quality educational resources in key senior high schools. The outcome of the *Zhongkao* is also crucial as it indirectly impacts their performance in the *Gaokao*, the nationwide college entrance examination taken in the 12th grade. The *Gaokao*, likewise, is highly demanding and carries significant weight in determining students’ access to quality higher education. Consequently, junior high school students are under immense pressure to excel in exams to secure admission to key senior high schools. This emphasis on exam scores is driven by the millennia-old Confucian value that diligence is a vital component of successful learning (Lee, 1999). Both students and teachers tend to prioritize exam scores over learning engagement and psychological well-being. However, it is essential to recognize that learning is a complex process that encompasses affective, behavioral, and cognitive outcomes (Christophel, 1990; Dağgöl, 2020; Derakhshan, 2022). Learners’ emotional experiences should not be disregarded, as students are likely to sense diverse emotions in the English learning process. These emotions have the potential to impact their learning engagement. In this context, we investigated the emotional experiences and L2 learning engagement of Chinese junior high school students from a Positive Psychology perspective, aiming to provide insights for English as a Foreign Language (EFL) teaching and promote the development of PP in SLA.

## Literature review

### Positive psychology in SLA

The emergence of PP can mainly be boiled down to the change in psychology’s focus during the 1970s. Rather than concentrating on human weaknesses and malfunctioning, the field shifted toward exploring human strengths and optimal functioning (Seligman and Csikszentmihalyi, 2000). This new perspective emphasized the positive factors that could promote individual and collective development and well-being. A significant milestone in PP occurred in 2000 when the journal *American Psychologist* published an influential special issue wherein Seligman and Csikszentmihalyi (2000) pinpointed the goals of PP and its three pillars. The first pillar involves exploiting positive emotions to enhance an individual’s positive life experience. The second pillar focuses on utilizing positive personality traits, such as virtues, strengths, and aptitudes. The third pillar emphasizes the exploitation of positive social institutions (e.g., democracy and education) that facilitate positive human development (Pluskota, 2014). Consequently, PP has become a prominent branch of psychology, emphasizing positive traits that contribute to individual and collective success and well-being. In recent years, scholars in related fields have increasingly recognized the significance of PP, and researchers in SLA have started to apply PP to their studies and teaching practices.

The emotional movement fostered by the huge progress of PP in SLA (MacIntyre and Mercer, 2014; Dewaele and Li, 2018; Prior, 2019) is rooted in the broaden-and-build theory (BBT), which distinguishes between positive and negative emotions (Fredrickson, 2001). According to this theory, positive emotional experiences broaden an individual’s short-term thought-action repertoire, leading to the accumulation and construction of long-term cognitive, motivational, physical, psychological, and social resources. Negative emotions, on the other hand, narrow these resources. The BBT also suggests that positive emotions can offset the pernicious impact produced by negative emotions (Fredrickson, 2001). MacIntyre and Gregersen (2012) asserted that positive emotions enhanced learners’ attention to new stimuli, thus facilitating language input absorption.

Another theory in educational psychology, the control-value theory (CVT), theoretically has also promoted the development of PP in SLA (Piniel and Albert, 2018; Han and Hyland, 2019; Shao et al., 2019; Li, 2020b). This theory focuses on emotions in academic situations, distinguishing different emotions based on valence, activation, and object focus (Pekrun, 2006). It systematically explains the antecedents and consequences of emotions, proposing that individuals’ control and value appraisals of academic achievement-related activities or outcomes serve as antecedents to academic emotions, which in turn have effects on academic activities and performance. While the BBT and the CVT share common points and compatibility, the latter provides a more comprehensive explanation of the emergence of emotions and their effects. Furthermore, the CVT accentuates the complex, systematic, and dynamic nature of the relationship between emotions and their influencing factors.

### Academic burnout

Burnout, a state of mental exhaustion, has been discovered in various professions (Schaufeli and Taris, 2005). Initially, research primarily focused on studying burnout in relation to occupational work (Maslach et al., 2001). The scope of studies later expanded to include student cohorts (Lin and Huang, 2012). As a result, researchers reconceptualized burnout and termed it “learning” or “academic” burnout (Lin and Huang, 2012), which is categorized into three aspects: exhaustion, cynicism, and inefficacy (Maslach et al., 2001). The concept of exhaustion is defined as a state of intense fatigue resulting from the depletion of emotional resources. Cynicism, on the other hand, is characterized by negative, indifferent, callous, or distancing attitudes toward learners’ study and individuals associated with their study. Inefficacy, or reduced efficacy in Li et al. (2021) term, refers to learners’ perception of incompetence or underachievement in study (Schaufeli and Salanova, 2007). Learners’ burnout can be induced by multiple factors, including social environment variables (e.g., economic level, peer competition, and employment prospects), personal internal variables (e.g., personality traits, self-efficacy, and time management), and learning environment variables (e.g., academic requirements, classroom space, and teacher communication) (Kim et al., 2018; Wang et al., 2019). Similar to other negative emotions, learning burnout can have detrimental effects on students, such as severe problems in mental and physical health (Schaufeli et al., 2002). Learners afflicted with burnout often exhibit a low sense of accomplishment, depersonalization, emotional fatigue, and so on (Lin and Huang, 2012).

As a significant topic in exploring the emotions of FL teachers (Hu and Wang, 2014), burnout extends beyond teachers to also affect students (Hu and Schaufeli, 2009). Like professionals in other fields, students engage emotionally and behaviorally in activities that can be seen as “work” from a psychological standpoint (Li et al., 2021). Particularly in structured and obligatory activities aimed at achieving specific goals, including class attendance, assignment completion, and test-taking, students are prone to experiencing burnout (Schaufeli and Taris, 2005). In the test-oriented context of Chinese EFL learning, burnout is more likely to occur among students, negatively impacting their learning efficacy and psychological well-being (Li, 2020a). Nevertheless, to our best knowledge, there is limited research on Chinese EFL learners’ burnout. Among the few existing studies, Wang (2013) investigated academic burnout in 184 college English learners, finding it to be a prevalent issue. English teachers were therefore urged to assist learners in creating practical plans, setting goals at different stages, evaluating performance, and implementing effective measures to sustain interest and optimize learning outcomes. Similarly, in a study by Li (2020a), 1,307 Chinese Year-2 senior high school students had a low level of burnout, and bivariate correlations were found between their emotional intelligence, enjoyment, anxiety, burnout, and English achievement. Results also showed that learners’ achievement emotions mediated the relationship between emotional intelligence and English achievement. Li et al. (2021) also confirmed that senior high school students’ learning burnout was at a low level. These findings underscore the importance of addressing burnout in English learners. In another recent study by Rong (2022), 465 college non-English majors were examined, revealing moderate levels of burnout and engagement among participants. Notably, different grades showed variations in burnout and engagement, with a negative correlation between them. Moreover, English learning burnout, including its three dimensions, negatively predicted English learning engagement, although the prediction was only partial.

Taken together, the current literature fails to completely reveal Chinese EFL learners’ burnout in their English learning journey and its interaction with other language learning variables. Additional empirical studies are required to examine these issues among various student groups.

### EFL learners’ academic engagement

Learning engagement is a concept that has been adapted for the field of education from job engagement. It is viewed as the antonym of learning burnout (Schaufeli et al., 2002). In the literature, learning engagement has been conceptualized in multiple ways (Derakhshan, 2021). Schaufeli et al. (2002) approached learning engagement from a psychological standpoint and defined it as the positive and enthusiastic attitude, abundant energy, resilience, and complete immersion displayed by individuals during the learning process. They conceptualized learning engagement as a trichotomous construct encompassing vigor (high energy levels, persistence, and intended effort in studying), dedication (a feeling of meaningfulness, passion, and challenge in learning), and absorption (full engrossment in learning). Fredricks et al. (2004) highlighted that learning engagement involved taking actions and differentiated this construct into three components: behavioral (time on task and participation), emotional (learners’ connection or disconnection from school and the people within the school), and cognitive (sustained attention and mental effort) engagement. Alternatively, Philp and Duchesne (2016) added social engagement to Fredricks et al. (2004) framework, considering the necessary interaction between learners, their peers, and instructors.

Academic engagement has consistently shown a range of positive effects on learning, even though it is conceptualized differently. Learners who are more engaged demonstrate better strategy application, self-control, satisfaction, and learning achievement (Wefald and Downey, 2009; Maricuțoiu and Sulea, 2019). Conversely, learners with higher levels of burnout struggle to engage in learning, despite having higher IQs (Akanni et al., 2019). A recent cross-sectional study by Wang et al. (2021) found that college students’ learning engagement and psychological capital were negatively associated with learning burnout during the COVID-19 pandemic. Moreover, the study found that learning engagement effectively reduced burnout.

Various terms have been used by researchers in FL teaching to interpret “engagement.” For instance, Guilloteaux and Dörnyei (2008) introduced the term “motivational behavior” to describe students’ engagement behaviors during English learning classes. Similarly, Dörnyei (2000) used the term “actional phase” to depict the scenario where learners actively participate in English learning tasks. It has been demonstrated in previous studies that students actively participating in in-class activities and interacting with their peers and teachers tend to achieve positive learning outcomes (Zhang, 2021). One survey conducted by Ding (2017) examined the English learning engagement of non-English major freshmen and sophomores. The results showed that students’ English learning engagement was generally below the average level. The study identified learning motivation, environment (school, family, and social environment), and self-efficacy as the key factors influencing college students’ engagement in English learning. However, more evidence is needed to draw robust conclusions regarding EFL learners’ engagement. This is evident from the mixed results of Lin (2020) study, which found moderate engagement among EFL learners in Taiwan, and Liu et al. (2022) findings indicating medium to high engagement among EFL learners in the Chinese mainland.

### Enjoyment in EFL learning

Scholarly interest in positive emotions (e.g., flow and enjoyment) has been growing (Oxford, 2015). As suggested by the three-dimensional taxonomy of achievement emotions in the CVT (i.e., valence, activation, and object focus), enjoyment is a positive, activating, and activity-based achievement emotion (Pekrun, 2006). In SLA, FLE is conceptualized as a trio-factorial construct consisting of FLE-Private, FLE-Teacher, and FLE-Atmosphere. FLE-Private refers to learners’ realization of progress, pride in pushing one’s limits, interest and novelty, positive changes, familiarity of the input, etc. FLE-Teacher is linked to teacher recognition and support, non-traditional pedagogical practices such as the use of multimedia, cultural activities, English songs, and role-play. FLE-Atmosphere pertains to teacher-controlled group activities with positive engagement from peers. FLE is a critical positive emotion that creates a secure psychological atmosphere for EFL learners and enhances their FL learning potentials (Dewaele and MacIntyre, 2014). FLE enables students to establish good interpersonal relationships in daily language learning and consistently progress toward their achievement goals (Chen et al., 2021). Therefore, learners’ enjoyment experience not only contributes to the improvement of their language proficiency but also helps maintain their psychological well-being.

Empirical research on FLE has consistently demonstrated its positive effects. For example, Dewaele and MacIntyre (2014) revealed that FLE enhanced FL learners’ thinking skills and reduced the negative emotional interference they experienced in a foreign culture. Likewise, Liu and Hong (2021) research on 709 students at the primary and secondary levels observed that when students enjoyed their language class, they became more attentive, active, and willing to allocate more time for learning English. In a study involving 320 senior high school English learners, Jin and Zhang (2018) unveiled a significant positive association between FLE and English performance. Li and Han (2022), in a questionnaire survey involving 348 Chinese non-English majors, found that FLE positively predicted English scores and perceived online learning achievement. These studies have considered FLE as an independent variable, demonstrating its direct positive impact. Additionally, there is also research that has investigated FLE as a mediating variable. Li (2020a) found that FLE acted as a mediator between learners’ emotional intelligence and English performance. Hu et al. (2022), based on a survey involving 388 English majors in a Chinese university, found that FLE acted as a mediator between growth mindsets and English learning proficiency. Lee et al. (2022) indicated that FLE mediated the link between informal digital English learning and willingness to communicate (WTC) among junior high school students in Hong Kong S.A.R. These findings further establish the recognized mediating role of FLE.

To date, FLE in the Chinese EFL teaching setting has been inadequately investigated in comparison to negative emotions in SLA. Therefore, further empirical evidence is necessary to unveil the positive emotional state of learners and its relation to other emotional constructs.

## Rationale, aims, and research questions of the present study

In the context of the “affective turn” transpiring in applied linguistic research, researchers in SLA have undertaken an extensive body of research worldwide. However, learner emotions, described as “the elephants in the room” by Swain (2013, p. 195), remain poorly studied and understood. Particularly in the Chinese EFL teaching context, where learners are obligated to learn a FL and pass numerous language tests from primary to tertiary levels, there is a greater emphasis on learners’ language learning and use rather than the complete learning process, which includes cognitive, affective, and behavioral changes. Consequently, there is a need for more empirical investigation into learners’ emotions to assist in our comprehension of their learning process. Furthermore, the majority of research on the emotions of Chinese EFL learners has primarily focused on college students and senior high school students, with only a few studies having explored junior high school students’ achievement emotions. Additionally, there is a lack of studies that have simultaneously examined the interrelationships among FLE, English learning burnout, and engagement. Taking into account these gaps and adopting the PP as its theoretical basis, the present study quantitatively surveyed Chinese junior high school students’ enjoyment, burnout, and engagement in English learning.

The study intended to address five research questions (RQs) as follows:

RQ1: What are the levels of burnout, engagement, and enjoyment among EFL learners?

RQ2: Are there gender/grade differences in learners’ burnout, engagement, and enjoyment levels?

RQ3: What are the correlations among learners’ burnout, engagement, and enjoyment?

RQ4: How well do the two achievement emotions (i.e., burnout and enjoyment) predict English learning engagement?

RQ5: Does enjoyment serve as a mediator between engagement and burnout?

## Research methods

### Participants and procedures

Participants included 387 junior high school students from three public secondary schools in the Chinese mainland. Among them, 42.12% (*n* = 163) were male and 57.88% (*n* = 224) were female. 171 of these students were in Grade 7 (44.19%), 138 in Grade 8 (35.66%), and 78 in Grade 9 (20.15%). The average age of the participants was 14.22 years (*SD* = 0.67). They had one EFL class (45 min) daily and had been studying English for at least 4 years since Grade 3 in elementary school. Before administering the questionnaire survey, we obtained the consent from the school administration, the headteacher, and the participants. Because students were prohibited from using mobile phones in school, the survey was conducted in pencil-and-paper form by the English teachers of each school. We allowed participants’ withdrawal from the survey at any time without any consequences.

### Instruments

#### Maslach Burnout Inventory-EFL Student (Chinese version)

This study utilized Li et al. (2021) 10-item Maslach Burnout Inventory-EFL Student (Chinese Version) to evaluate the level of burnout among students in their English learning. The inventory consists of three sub-scales: Exhaustion (4 items), Cynicism (3 items), and Reduced Efficacy (3 items). Examples of items from each sub-scale include “I feel emotionally drained by my English studies” for Exhaustion, “I have become less enthusiastic about my English studies” for Cynicism, and “During English class I do not feel confident that I am effective in getting things done” for Reduced Efficacy. The participants responded to the questionnaire on a 5-point Likert scale ranging from 1 (strongly disagree) to 5 (strongly agree). A lower score suggests a lower burnout level. The Cronbach’s alpha coefficient for the entire scale was 0.901, demonstrating an exceptionally high level of reliability that surpassed the minimum cutoff point of 0.7.

#### Utrecht Work Engagement Scale-Student (UWES-S, Chinese version)

The Chinese version of UWES-S, as translated by Fang et al. (2008), was utilized to measure students’ engagement in English learning. The tri-factorial 17-item UWES-S scale comprises of three dimensions: Vigor (6 items), Dedication (5 items), and Absorption (6 items). Example items that represent each dimension include “When I get up in the morning, I feel like going to class” for Vigor, “My study inspires me” for Dedication, and “Time flies when I am studying” for Absorption. To gauge their agreement with each statement, the respondents rated them on a 7-point Likert scale, ranging from 1 (never) to 7 (always). A lower score indicates a lower level of engagement. Furthermore, the questionnaire demonstrated high reliability (*α* = 0.935) in this study.

#### Chinese version of the Foreign Language Enjoyment Scale

The modified 11-item CFLES (Li et al., 2018) was adopted to evaluate learners’ FLE. This scale consists of 11 items with 3 sub-scales: FLE-Private (5 items), FLE-Teacher (3 items), and FLE-Atmosphere (3 items). Example items include “Learning English is fun” (FLE-Private), “The English teacher is encouraging” (FLE-Teacher), and “There is a good atmosphere in Learning English” (FLE-Atmosphere). Students were required to report their FLE on a 5-point Likert scale ranging from 1 (strongly disagree) and 5 (strongly agree). A lower score indicates less enjoyment. CFLES was also reported to be highly reliable (*α* = 0.939).

### Data collection and analysis

In June 2023, a questionnaire survey was conducted using convenience sampling to gather data. Paper-and-pen questionnaires were administered to the respondents, and the data was manually inputted into Excel sheets. Prior to data entry, the questionnaires were thoroughly examined to ensure all responses were complete, and incomplete cases were removed from the analysis. Descriptive analyses were performed to illustrate the levels of learners’ burnout, engagement, and enjoyment (RQ1). Normality tests were conducted to determine the appropriate statistical procedures for subsequent analyses. The results of normality tests determined whether parametric or non-parametric analyses were conducted to examine gender and grade differences (RQ2). Correlation analyses and regression analyses were conducted to explore the relationships among the three variables (RQ3 and RQ4). To answer the last RQ, we applied the bias-corrected bootstrapping method within a 95% confidence interval. The data were analyzed using SPSSAU (an online software).^1^ The statistical significance was set at the *p* < 0.05 level.

## Research results

After reviewing all participants’ responses and eliminating incomplete ones, we retained 368 (95.09%) valid responses. The normality of the data was assessed using the Kolmogorov–Smirnov method due to the sample size exceeding 100 (Drezner et al., 2010). The statistics presented in Table 1 reveal significant *d*-values (*p* < 0.05), indicating rejection of the null hypothesis and non-normal distribution of the data. Therefore, non-parametric analyses were conducted in subsequent statistical procedures.

**Table 1:** Descriptive statistics and results of normality tests (*n* = 368).

Variables	Possible range	Minimum	Maximum	Mean	*SD*	Kolmogorov–Smirnov test	
						*d*	*p*
Burnout	10–50	10	46	22.12	7.473	0.159	0.000**
Enjoyment	11–55	19	55	44.084	8.67	0.175	0.000**
Engagement	17–119	51	119	97.378	21.92	0.257	0.000**

SD, standard deviation.**p < 0.01.

### Students’ burnout, engagement, and enjoyment levels

Table 1 provides the descriptive statistics. As can be seen, the mean scores of enjoyment (*M* = 44.084, *SD* = 8.67) and engagement (*M* = 97.378, *SD* = 21.92) were observed to be high, whereas the mean score of burnout was found to be low (*M* = 22.12, *SD* = 7.473). Thus, it can be inferred that junior high school students experienced minimal burnout but enjoyed their studies and were highly engaged in learning English.

### Gender and grade differences in the three variables

Non-parametric analyses were run to inquire into whether gender and grade differences existed in learners’ burnout, enjoyment, and engagement, as previously mentioned, due to the non-normally distributed data. According to Pallant (2020), the non-parametric rank test assesses the variability of Y across various groups of X. When X has two groups, the Mann–Whitney’s test is conducted, whereas the Kruskal-Wallis test is suitable when X has more than two groups. Table 2 indicates that burnout did not exhibit any gender differences (*p* > 0.05). However, significant gender disparities were observed regarding learning enjoyment and engagement (*p* < 0.05).

**Table 2:** Gender differences.

Variables	Gender (median)		*u* (Mann–Whitney’s test)	*z*	*p*
	1.0 (*n* = 148)	2.0 (*n* = 220)			
Burnout	24.5	20	15,258	−1.025	0.305
Enjoyment	45	47.5	14012.5	−2.27	0.023*
Engagement	102	105	14,184	−2.105	0.035*

1 = male; 2 = female.*p < 0.05.

**Figure 1:**
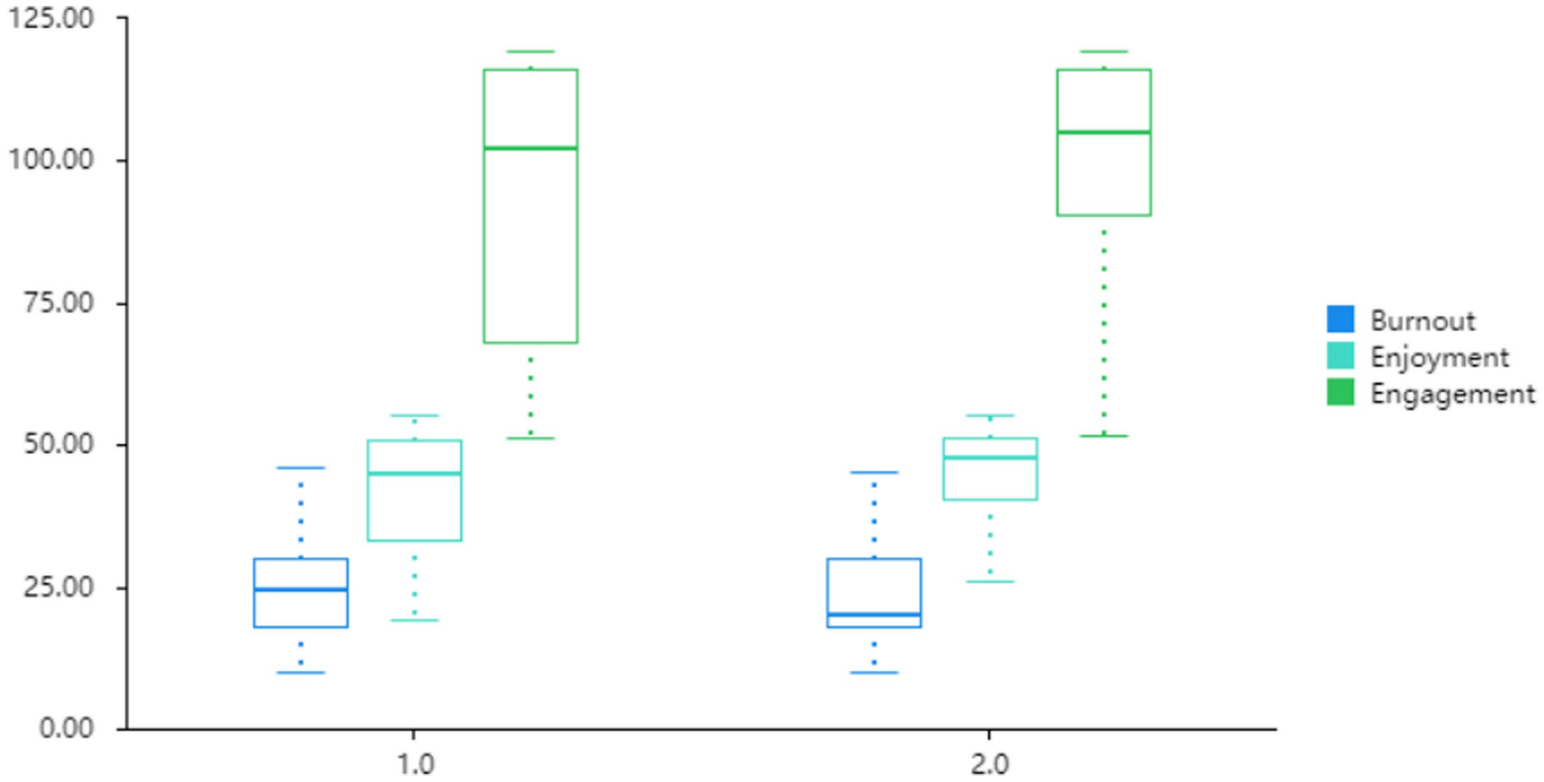
The box plot illustrating gender differences. 1 = male; 2 = female.

Figure 1 presents a box plot depicting the gender differences in learners’ burnout, enjoyment, and engagement levels. Typically, a positively skewed box plot is obtained when the median-maximum distance is greater than the median-minimum distance. Otherwise, a negatively skewed box plot is observed. In relation to this, Figure 1 reveals that male students’ median burnout level was higher than that of females. Furthermore, the median engagement and enjoyment levels for male students were lower than those of their female counterparts. These findings indicate that male students experienced higher burnout levels and reduced enjoyment and engagement in English learning compared to females. Consequently, the results illustrated in Figure 1 align with those presented in Table 2.

Table 3 shows the variations in learners’ burnout, engagement, and enjoyment across different grades. The results indicate significant grade differences in these three variables (*p* < 0.01).

**Table 3:** Grade differences.

Variables	Grade (median)			*H*-value (Kruskal-Wallis test)	*p*
	1.0 (*n* = 162)	2.0 (*n* = 132)	3.0 (*n* = 74)		
Burnout	19	21	30	77.961	0.000**
Enjoyment	49	46	34	61.042	0.000**
Engagement	109	105	72.5	55.003	0.000**

1 = Grade 7; 2 = Grade 8; 3 = Grade 9.**p < 0.01.

*Post-hoc* test reveals significant grade differences in burnout (see Table 4). Specifically, Grade 9 showed the highest level of burnout, followed by Grade 8, and Grade 7 exhibited the lowest level of burnout. Additionally, although Grade 7 demonstrated greater enjoyment and learning engagement compared to Grade 8, the difference was found to be non-significant. Conversely, Grade 9 exhibited significantly lower levels of enjoyment compared to both Grade 7 and Grade 8. These findings suggest that as students progressed through different grade levels, their burnout levels increased while their enjoyment and learning engagement decreased.

**Table 4:** *Post hoc* comparisons for grade differences.

Variables	Grade	Grade	Median	(J) Median	Difference (I-J)	*p*
Burnout	1	2	19	21	−2	0.005**
	1	3	19	30	−11	0.000**
	2	3	21	30	−9	0.000**
Enjoyment	1	2	49	46	3	0.137
	1	3	49	34	15	0.000**
	2	3	46	34	12	0.000**
Engagement	1	2	109	105	4	0.303
	1	3	109	72.5	36.5	0.000**
	2	3	105	72.5	32.5	0.000**

1 = Grade 7; 2 = Grade 8; 3 = Grade 9.**p < 0.01.

**Figure 2:**
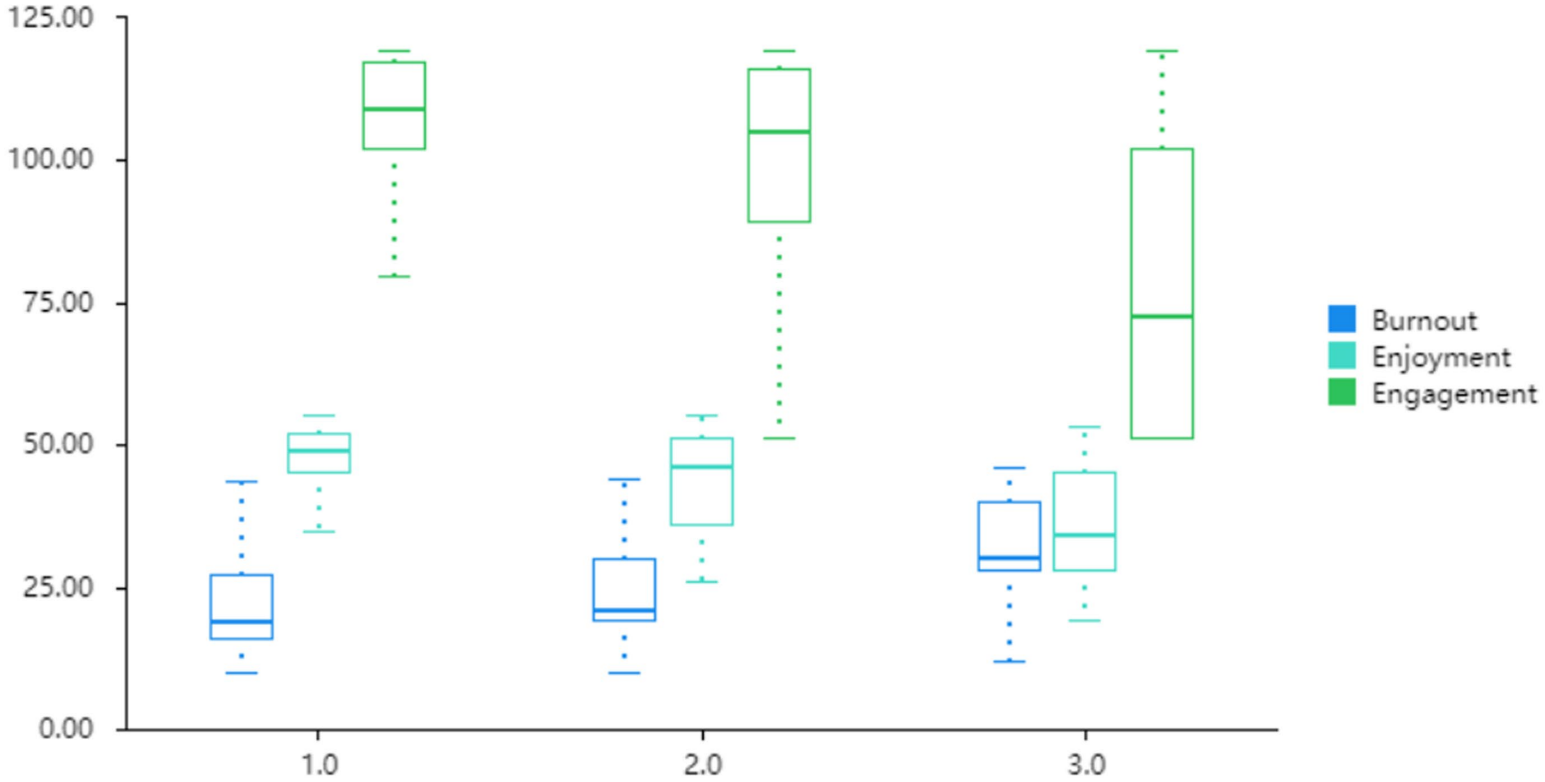
The box plot illustrating grade differences. 1 = Grade 7; 2 = Grade 8; 3 = Grade 9.

It is noticeable in Figure 2 that, from Grade 7 to Grade 9, students’ median burnout level showed an upward trend, indicating that junior high school students experienced the highest burnout in Grade 9. In contrast, learners’ median enjoyment and engagement levels exhibited a downward trend, suggesting that they enjoyed their studies the least and engaged the least in Grade 9. The trends observed in the burnout, enjoyment, and engagement levels are consistent with the patterns indicated in Table 4.

### Correlations among burnout, engagement, and enjoyment levels

To examine the interplay between learners’ burnout, engagement, and enjoyment, we conducted Pearson correlation analyses. The results in Table 5 revealed significant negative correlations between burnout and both enjoyment (*r* = −0.837, *p* < 0.01) and engagement (*r* = −0.790, *p* < 0.01). Conversely, enjoyment and engagement showed a strong positive correlation (*r* = 0.960, *p* < 0.01). These results reveal that learners who felt less burnout were likely to sense more enjoyment and engage themselves more in the learning activities.

**Table 5:** Bivariate correlations (*n* = 368).

	Burnout	Enjoyment	Engagement
Burnout	1		
Enjoyment	−0.837**	1	
Engagement	−0.790**	0.960**	1

**p < 0.01.

### Predictive effects of enjoyment and burnout on engagement

We conducted multiple regression analysis employing the enter method to examine the relationship between the dependent variable (engagement) and two independent variables (enjoyment and burnout). Tolerance values for enjoyment and burnout were found to be 0.299 and 0.186 respectively, indicating no collinearity between these variables. In addition, the residuals of the regression model met the standards for normality, linearity, homoscedasticity, and independence as outlined by Pallant (2020). The results presented in Table 6 demonstrated a significant regression equation (*F* = 2168.701, *p* < 0.001). Specifically, enjoyment displayed a significant and positive prediction of English learning engagement (*β* = 0.498, *p* < 0.001). On the other hand, burnout negatively predicted learning engagement (*β* = −0.046), but this predictive effect was not significant (*p* > 0.05). When combined, the two independent variables accounted for 49.2% of the variance in learning engagement (adjusted *R*^2^ = 0.492), suggesting that enjoyment emerged as the main predictor of English learning engagement.

**Table 6:** Results of multiple regression analysis.

	B	*SE*	Beta	*t*	*Sig.*	Tolerance	VIF	*F*	Adjusted *R*^2^
Constant	−16.727	4.467		−3.744	0.000			2168.701	0.492
Enjoyment	2.524	0.067	0.498	37.459	0.000	0.299	3.339		
Burnout	−0.118	0.069	−0.046	−1.709	0.088	0.186	5.376		

### Enjoyment as a mediator between engagement and burnout

To examine the mediation effect of enjoyment, bootstrapping was utilized on the basis of 5,000 samples. According to Hayes (2009), significant indirect effects would be indicated if the 95% confidence interval between the lower bound and upper bound did not include zero. The effect size can be categorized as small if its absolute value is less than 0.1 and as large if it is equal to or greater than 0.3 (Gignac and Szodorai, 2016).

The findings of this study, as demonstrated in Table 7, indicate several important relationships between the variables examined. First, regarding the direct relationship between engagement and burnout, a negative and small effect was observed (effect size = −0.067), but this direct effect was statistically non-significant (*p* > 0.05). In contrast, the indirect effect of engagement on burnout, mediated by enjoyment, was found to be both negative, significant and large (effect size = −0.373, *SE* = 0.097, 95% C. I. = [−1.151, −0.774], *p* < 0.05). In addition, the total effect of engagement on burnout was also found to be significant (effect size = −0.440, *p* < 0.05). These suggest that enjoyment fully mediated the association between engagement and burnout. Furthermore, the findings reveal a positive and significant association between engagement and enjoyment (effect size = 0.380, *p* < 0.05). It is also found that enjoyment had a negative and significant correlation with burnout (effect size = −0.981, *p* < 0.05). Furthermore, a positive and significant association between engagement and enjoyment was obtained, whereas enjoyment was negatively and significantly linked to burnout. Overall, these results highlight the intricate relationship between engagement, enjoyment, and burnout.

**Table 7:** Summary of the mediating effect test (*n* = 368).

c	a	b	a*b				c’
			Size	Boot *SE*	*z*	(95% Boot C.I.)	
−0.440**	0.380**	−0.981**	−0.373**	0.097	−3.839	−1.151 ~ −0.774	−0.067

a means the direct effect of engagement on enjoyment; b denotes the direct effect of enjoyment on burnout; a*b means the mediation effect of enjoyment; c signifies the total effect; c’ refers to the direct effect of engagement on burnout; SE means standard deviation; C.I. denotes confidence interval. **p < 0.01.

## Discussion

This study endeavored to examine Chinese junior high school students’ burnout, enjoyment, and engagement in English learning. By reviewing past research and relevant theories, this section comprehensively analyzes and interprets the results.

Our findings indicate that participants experienced minimal burnout and instead actively engaged themselves in the English learning process, displaying a genuine enjoyment for it. First, we discovered that EFL learners displayed a high degree of enjoyment, resonating with Jiang (2023) study on Chinese junior high school students. Commencing English studies in Grade 3, Chinese EFL learners are commonly exposed to fragmented English expressions without a systematic approach to language acquisition. Consequently, language learning at the secondary level might still be a novel experience for them. Nonetheless, their curiosity and interest in the English language drives them to manifest joyfulness during the learning process (Isikman et al., 2016). However, to provide evidence, more in-depth research ought to be conducted. Second, the results of the present study agree with Li (2020a) and Li et al. (2021) observation that Chinese senior high school students perceived little burnout in English learning. The findings of the present study are, however, inconsistent with Rong (2022) conclusion that college non-English majors experienced a moderate level of English learning burnout and engagement. This difference might be explained by the fact that in the Chinese EFL teaching context, students at the secondary level tend to be faced with more academic pressure in English learning than those in higher education. High school students, in general, are obligated to take entrance examinations (with the English tests occupying an important place) and fulfill the expectations of their teachers, parents, and peers. As indicated by Xiang et al. (2022), the academic pressure acts as a strong motivation for learning and is negatively associated with learning burnout. High learning motivation facilitates the use of effective cognitive strategies, enhances stress management, and reduces dropout rates (Yang, 2004). On the other hand, college students (especially non-English majors) are less burdened with academic pressure in learning English, which may result in a decline in learning engagement and an increase in learners’ burnout experience. The results confirm the CVT, which postulates that learners who perceive learning activities or outcomes to hold intrinsic and extrinsic value and believe they have control over these activities or outcomes experience more positive emotions and greater engagement in learning (Pekrun, 2006).

Notably, we observed gender and grade disparities in relation to learners’ burnout, engagement, and enjoyment. Specifically, male students demonstrated significantly lower levels of enjoyment and engagement compared to their female counterparts, while their level of burnout was comparable. There were also noteworthy differences in burnout levels among students of different grades, with an upward trend. However, there was no significant difference in enjoyment and engagement levels between Grade 7 and Grade 8. These findings partially concur with those of Rong (2022) but diverge from those of Guo (2021). The gender differences in enjoyment and engagement could be ascribed to gender intensification or socialization pressures as discussed by Hill and Lynch (1983). It is possible that female students exhibit enhanced language learning abilities, outperforming male students, and displaying greater motivation to learn. Regarding the grade differences, it is imperative to consider the Chinese EFL learning context. Middle school students in higher grades may experience greater exhaustion due to their participation in numerous English tests, despite being aware of the importance of English learning. Gradually, from Grade 7 to Grade 9, they may encounter increasing burnout, diminishing enjoyment in English learning, and reduced engagement in learning activities. However, it is worth noting that these explanations require further empirical validation.

Correlation analyses identified strong and negative correlations between burnout and engagement and enjoyment. That is, students feeling more exhaustion, cynicism, or reduced efficiency in English learning had less enjoyment and were less engaged in learning. This finding partially dovetails with Rong (2022) research, which also found a strong and negative association between burnout and engagement among college EFL learners. This proves Schaufeli et al. (2002) argument that engagement is a theoretical opposite to burnout. While the current study and Rong (2022) research differ in terms of their samples, they do support the notion that enhancing students’ academic engagement is advantageous in mitigating burnout (Wang et al., 2021). Furthermore, the present study partially corroborates the findings of Li (2020a), which observed a moderate and negative relationship between burnout and enjoyment. These findings also lend empirical support to the CVT (Pekrun, 2006), which categorizes burnout and enjoyment as opposing emotions. Additionally, we uncovered a strong positive correlation between engagement and enjoyment, confirming the findings of Guo (2021) and suggesting that FLE plays a crucial role in sustaining learners’ engagement in learning (Mercer and Dörnyei, 2020).

Concurring with the extant literature (e.g., Hosseini et al., 2022; Tsang and Dewaele, 2023), our research provided evidence that FLE was a significant and positive predictor of L2 learning engagement. This positive relationship could be explained by the BBT, which posits that positive emotions, such as enjoyment, can broaden learners’ thought-activity repertory and build their personal resources and affective flexibility (Fredrickson, 2001). Moreover, Dewaele and Macintyre (2014) suggest that positive emotions enhance learners’ self-discovery, acquisition of novel experiences, and learning effectiveness. Considering that the participants of our study were junior high school students, who might still be exploring English learning as a new territory, it is likely that they would experience more positive emotions and engage more in the learning process.

A more valuable finding in this study consists in that enjoyment served as a full mediator between engagement and burnout. Simply put, enhancing students’ engagement in language learning alone does not necessarily decrease their burnout. Rather, when learners enjoy language learning, they tend to actively engage themselves in learning English. This finding also validates the BBT, which posits that positive emotional experiences, including enjoyment, potentially expand learners’ thought-action repertoires and counteract the debilitating effects of negative emotional states, like burnout (Fredrickson, 2001).

## Conclusion

This study has made unique contributions to the literature in SLA by revealing learners’ levels of burnout, engagement, and enjoyment, as well as exploring gender and grade differences and their interrelationships. The findings have important implications. Theoretically, it has provided empirical evidence for the applicability of the BBT and the CVT in SLA. Especially, enjoyment had a substantial predictive impact on engagement and also played a mediating role between engagement and burnout. Pedagogically, language teachers should prioritize the consideration of EFL learners’ gender and grade differences. Moreover, teachers should create positive learning environments for students because they may have a range of emotions while learning a FL. To address the challenges or negative experiences that may arise, students need to be made aware of their own strengths and encouraged to make full use of these advantages. By finding positive value in these challenges and transforming them into positive experiences, students may become more actively engaged in their learning. Additionally, enhancing students’ emotional awareness and regulation can provide long-term benefits in handling complex, diverse, and dynamic emotions. This not only improves their well-being during the learning process but also promotes their academic development.

Despite these implications, the present study is constrained by several limitations. Firstly, due to convenience sampling, the representativeness of participating students (only from three schools) was not sufficiently guaranteed, which may undermine the generalizability of the results to other student cohorts. Secondly, the sample size of this study, especially that of Grade 9, was limited, which might compromise the validity of cross-grade comparisons. Future studies should consider a more representative and larger sample. Thirdly, this study involved a cross-sectional research design and failed to reveal the dynamicity of learners’ burnout, engagement, and enjoyment. Whether the changes in these three variables will present a different story in terms of their interrelationships remains to be investigated. Therefore, a longitudinal research design is more desirable to unveil the dynamicity. Lastly, this study solely used a quantitative research method, which does not provide a comprehensive picture of the examined issue. To address this limitation, a mixed research method would be more robust.

Beyond addressing the above-mentioned limitations, researchers in SLA can expand the research scope. For example, it is advisable to conduct research involving L2 learner populations from multiple countries or regions, in order to compare and contrast their emotional experiences and learning engagement. Investigating whether cultural backgrounds shape learners’ achievement emotions and learning engagement is still needed. Moreover, researchers can explore learners’ emotional states and engagement in various L2 skills, including listening, speaking, reading, writing, translating, and interpreting.

## Data availability statement

The raw data supporting the conclusions of this article will be made available by the authors, without undue reservation. Requests to access these datasets should be directed to corresponding author via wangjian@guc.edu.cn.

## Ethics statement

The studies involving humans were approved by Human Research Ethics Committee of Panzhihua University. The studies were conducted in accordance with the local legislation and institutional requirements. Written informed consent for participation in this study was provided by the participants' legal guardians/next of kin.

## Author contributions

XZ: Writing - original draft, Writing - review & editing, Conceptualization. JW: Investigation, Methodology, Writing - review & editing, Software. XK: Investigation, Writing - review & editing.

## Footnotes

^1^ https://spssau.com/indexs.html
